# Supplementary material for: Knowledge, attitude and management of hearing screening in children among family physicians in the Kingdom of Saudi Arabia
Source: PLoS One. 2021 Aug 31;16(8):e0256647. doi: 10.1371/journal.pone.0256647 (PMC8407574; doi:10.1371/journal.pone.0256647)
Supplement: Attachment — Submitted filename: Response to Reviewers.docx [file pone.0256647.s002.docx]

| Reviewer Number | Original comments of the reviewer | Reply by the author(s) | Changes done on page number and line number |
| --- | --- | --- | --- |
| Journal Requirements | Please ensure that your manuscript meets PLOS ONE's style requirements, including those for file naming. | The changes have been done |  |
| Journal Requirements | Please include additional information regarding the survey or questionnaire used in the study and  ensure that you have provided sufficient details that others could replicate the analyses. For instance,  if you developed the survey or questionnaire as part of this study and it is not under a copyright more restrictive than CC-BY, please include a copy, in both the original language and English, as  Supporting Information. If the questionnaire is published, please provide a citation to the (1)  questionnaire and/or(2) original publication associated with the questionnaire. | More details regarding the questionnaire have been added “The data were collected using a self-reported questionnaire. The questionnaire was adapted from one developed by Moeller, White and Shisler in 2006,^21^ including 25 questions divided on two main sections: the first section includes 6 questions on the demographic data and participants’ clinical backgrounds, in particular specialty, years of experience, gender, age and clinical setting. The respondents were also inquired about the number of infants referred to them after neonatal hearing screening, as well as the approximately number of children with permanent sensorineural hearing impairment they checked for the last three years. The second section includes a set of 19 questions assessing participants’ knowledge, attitudes and practices regarding aspect of audiology, involving diagnostic evaluation, hearing screening, and hearing loss management. The questions could differ in the type of answer, by way of multiple choice or short answers. Additionally, items of the scale will be formed to target the aims of this study and measure the expected outcomes comprehensively. For the purpose of assuring that the questionnaire of this research is proportionate with predictable results, similar KAP questionnaires from the published literature were studied very carefully and used as references. As a last step to validate our questionnaire, a pilot sample comprising 35 participants was used for investigating the coherence and comprehension of all questions. | Lines 170-178 on page 8 of the unclean copy |
| Journal Requirements | Thank you for stating in the text of your manuscript "This study was approved by the Institutional Review Board at King Fahad Medical City Riyadh, KSA (IRB000010471). Before enrolling potential participants, researchers provided information about the purpose and significance of the study. Furthermore, researchers emphasized voluntary participation. Moreover, all participants will be informed about anonymity, confidentiality issues and the option of voluntary termination at any time. At the beginning of the questionnaire, the participants were asked to give their consent before moving forward in filling the questionnaire." Please also add this information to your ethics statement in the online submission form. | The suggestion was considered, information was uploaded. |  |
| 1 | I would suggest including in the title the word children the Knowledge, Attitude, and Practice of Hearing Screening (in children) and Management among Family Physicians in Kingdom of Saudi Arabia, as the investigation relates to Hearing screening in children When speaking about language, it should be stressed we spoken language is considered (mostly in opposition to sign language or as a complement to sign language?) | The suggestion was done. | Title Line 1 on page 1of the unclean copy |
| 1 | Methods: the total number of family physicians in the KSA that could have been enrolled -that eligible- should be mentioned | Based on the information provided by the Saudi commotion for health specialties, the total number of family physicians 2700; however, we cannot get their contact information from representative authorities, so we follow networking and snowballing sampling technic and are able to target 300 and recruit 133 family physician. | Lines 35-40 on pages 2 of the unclean copy |
| 1 | 75: ....it is done before the baby is discharged home | Corrected | Line 78 on page 4 of the unclean copy |
| 1 | 127: They are also responsible for the identification of hearing loss and referring patients and discussing with parents the management plan; How exactly are family doctors responsible for the identification of the hearing loss? What is their role? do they perform diagnostic tests? are all "refer" newborns referred do the family doctors for further diagnostic tests? | In Saudi Arabia, all newborns should be screened for hearing impairment in the hospital before discharge. However, the Ministry of Health statistics showed that screening tests do not cover 30% of newborns.26 As a result, there is an emphasis from the Ministry of Health that the family doctor in primary health care should check with the family if their child undergoes hearing screening in the hospital before discharge. In case the screening not done, the family doctor will refer the child to conduct hearing screening in the hospital as soon as possible. | Lines 127-134 on page 6 of the unclean copy |
| 1 | 139: (related to 35) How many family medicine physicians were invited to participate in the study, and what is the universe of family physicians in the KSA? | Based on the information provided by the Saudi commotion for health specialties, the total number of family physicians 2700; however, we cannot get their contact information from representative authorities, so we follow networking and snowballing sampling technic and are able to target 300 and recruit 133 family physician | Lines 150-154 on page 7 of the unclean copy |
| 1 | 185:. Conversely, around 51% (68/113) of participants have screened children for hearing loss and around 60% (80/113) were able to diagnose one or more children with hearing loss during their last five years of experience. How did they screened: did they participate in maternity-based screening? and how did they diagnose hearing loss? do they performed diagnostic ABR or other audiological tests? | Conversely, around 51% (68/113) of participants have screened children for hearing loss, during the child's visit to the well-baby clinic to receive vaccinations and to assess his development, the family doctor asks the parents whether their child underwent a hearing screening test in the hospital. If not, the child will be referred to the hospital for the necessary evaluation according to the protocol followed in this institution and a. Around 60% (80/113) were able to diagnose one or more children with hearing loss during their last five years of experience (Table 1). | Lines 202-206 on page 9 of the unclean copy |
| 1 | 209: Concerning #CConcerning | Corrected | Line 228 on page 10 of the unclean copy |
| 1 | 221: figure 2 demonstrate the referral options followed by the family physician for children diagnosed with a confirmed hearing loss. Who confirmed the hearing loss? The flowchart of the various stages from screening to intervention proposed in the KSA program should be explained. It seems that after the established diagnose the child goes back to the family doctor and is then referred for audiological intervention? | The family doctor will ask parents if their child had undergone a hearing screening test at birth or if the parents noticed any symptoms on the child, the child is referred to the audiologist for the hearing test. When the child returns for follow-up, the reports are revised by the family doctor; if the result is abnormal, the child is then referred according to the type or cause of hearing loss to the appropriate specialty | Line 241 -245 on page 11 of the unclean copy |
| 1 | 322-324: this statement is a double negative. Does it mean that children with cochlear implant or hearing aids do well without speech therapy or hearing rehabilitation? | The sentences were modified as instructed by the reviewer. |  |
| 1 | 335: unilateral hearing loss or mild hearing loss impairs communication/hearing in noise (which is very prevalent in school/ kindergarten environment )as well as localization | The sentence was modified as instructed by the reviewer. |  |
| 2 | The article is quite interesting and practical. It has the potential to help create awareness in the population of family physicians, on the importance of screening and diagnosis of hearing loss. It would be interesting to dive deeper into this question: if physicians have good knowledge of the assessment, but often fail to complete the diagnosis, why does this occur? Is it lack time or attention? Which programs or interventions could the authors suggest improving this intention action gap | The following paragraph has been added to address the requested information by the reviewer” For instance, the responses to the study questionnaire revealed family physicians have good knowledge of the assessment, but often fail to properly reach a diagnosis. Although that family practitioners are the most- in demand medical subspeciality, based on the report from a physician recruitment institution called Merritt Hawkins in 2019 45, a family clinician may rush through his appointments, spend less time with his patients, and ultimately not complete diagnostic procedures and refer the patient to the relevant specialists. Moreover, the family physician takes professional responsibility for managing comprehensively undifferentiated health issues and is committed to clients irrespective of their sicknesses or impacted body system. This broad scope of practice may contribute to a lack of attention focused on developing diagnostic skills for all aspects of the practice, including audiological diagnostics. Several trainings and programs in diagnosing hearing impairment, interpreting test results, intervention techniques, and parents counseling were suggested by the study’s authors and participants to improve this intention- action gap of family medicine practice in the KSA.” | Lines 368-381 pages 16 of the unclean copy |
