# Supplementary material for: Knowledge, attitude and management of hearing screening in children among family physicians in the Kingdom of Saudi Arabia
Source: PLoS One. 2021 Aug 31;16(8):e0256647. doi: 10.1371/journal.pone.0256647 (PMC8407574; doi:10.1371/journal.pone.0256647)
Supplement: Attachment — Submitted filename: response to reviewers.docx [file pone.0256647.s004.docx]

| comment | Action | page | Line |
| --- | --- | --- | --- |
| The title should be corrected to: Knowledge, Attitude and Management of Hearing Screening in children among Family Physicians in the Kingdom of Saudi Arabia | The Suggested comment has been considered | Page: 1 | 1-3 |
| The sentence in Abstract Line 39 and Material and Methods line 157 is redundant to the previous sentence in the same sections and should be removed. Instead, it should be rewritten and sent to the end of the manuscript as a limitation of the study (133 out of 2700 family physicians). | The Suggested comment has been considered | Page: 2  Page: 18 | 41-42  402-406 |
| Correct grammar in Abstract line 27: "to develop normal spoking 28 language and academic skills during childhood". | The Suggested comment has been considered | Page: 2 | 30-31 |
| The new sequencing of the references in the revised main text is now difficult to follow, with numbers in red and black side-to-side | The Suggested comment has been considered |  |  |
